# Rapid ethnographic assessment for potential anti-malarial mass drug administration in an outbreak area of Santo Domingo, Dominican Republic

**DOI:** 10.1186/s12936-021-03594-5

**Published:** 2021-02-08

**Authors:** Hunter Keys, Keyla Ureña, Jhefres Reyes, Kevin Bardosh, Christopher Pell, Jose Puello, Stephen Blount, Gregory S. Noland

**Affiliations:** 1 Department of Anthropology, University of Amsterdam, Building B-REC B 8.01, Nieuwe Achtergracht 166, 1018 WV Amsterdam, The Netherlands; 2 Centro de Prevención y Control de Enfermedades Transmitidas por Vectores y Zoonosis (CECOVEZ), Av. Juan Pablo Duarte No. 269, 10301 Santo Domingo, Dominican Republic; 3 Colegio de Abogados de la Republica Dominicana, Santo Domingo, Dominican Republic; 4 Center for One Health Research, School of Public Health, University of Washington, Washington, USA; 5 Amsterdam Institute for Global Health and Development (AIGHD), Centre for Social Science and Global Health, University of Amsterdam, Amsterdam, The Netherlands; 6 Department of Global Health, Amsterdam University Medical Centers, Academic Medical Center, University of Amsterdam, Amsterdam, The Netherlands; 7 The Carter Center, 453 Freedom Parkway, Atlanta, GA 30307 USA

**Keywords:** Rapid ethnographic assessment, Mixed methods, Malaria, Mass drug administration, Dominican Republic

## Abstract

**Background:**

In the Dominican Republic, a recent outbreak of malaria in the capital, Santo Domingo, threatens efforts to eliminate the disease. Mass drug administration (MDA) has been proposed as one strategy to reduce transmission. The success of MDA is contingent upon high levels of acceptance among the target population. To inform the design of future MDA campaigns, this rapid ethnographic assessment examined malaria-related knowledge and attitudes toward malaria MDA among residents of a transmission focus in Santo Domingo.

**Methods:**

In October 2019, a rapid ethnographic assessment was conducted in the Los Tres Brazos transmission focus, which had not previously received MDA. National malaria programme staff conducted 61 structured interviews with key informants, recorded observations, and held 72 informal conversations. Using a grounded theory approach, data were analysed during three workshop sessions with research team members.

**Results:**

Among those who had heard of malaria in the structured interviews (n = 39/61; 64%), understanding of the disease was largely based on personal experience from past outbreaks or through word-of-mouth. Community health workers (*promotores*) were trusted for health information and malaria diagnosis more so than professional clinicians. No participant (0%) was familiar with malaria MDA. After learning about MDA, almost all study participants (92%) said that they would participate, seeing it as a way to care for their community. Reasons for not participating in future MDA included not trusting drug administrators, feeling reluctant to take unprescribed medicine, and fear of missing work. Additional identified challenges to MDA included reaching specific demographic groups, disseminating effective MDA campaign messages, and managing misinformation and political influence.

**Conclusion:**

Residents appear accepting of MDA despite a lack of prior familiarity. Successful MDA will depend on several factors: fostering relationships among community-based health workers, clinicians, community leaders, and others; developing clear health messages that use local terms and spreading them through a variety of media and social networks; and contextualizing MDA as part of a broader effort to promote community health.

## Background

The Dominican Republic (pop. 10.6 million) and Haiti (pop. 10.8 million) share the island of Hispaniola, the only remaining malaria-endemic island in the Caribbean [1, 2]. All locally transmitted cases on Hispaniola are caused by *Plasmodium falciparum*. Transmission by *Anopheles albimanus* mosquitoes occurs year-round with minor seasonal peaks typically observed in the Dominican Republic from June-July and January-December. First-line treatment for uncomplicated malaria is chloroquine-primaquine. Both countries have committed to malaria elimination [3].

In the Dominican Republic, recent outbreaks in the capital, Santo Domingo, have signaled a major shift from rural to urban transmission [4, 5]. Historically, malaria was most abundant along the Haiti border and in agricultural areas populated by migrant workers [6]. However a recent study indicates the absence of malaria from such rural areas [5]. The Santo Domingo metropolitan area (pop. 2.9 million) is comprised of the National District and Santo Domingo East, Santo Domingo West, and Santo Domingo North municipalities. Since 2014, this area has accounted for the majority of reported malaria cases nationwide. In 2019, 85% of the 1301 autochthonous cases occurred in the capital area. In comparison, no more than 65 cases annually were reported in 2011–2013 (< 7% of all cases nationally). Concurrently, imported cases from Haiti fell from 565 in 2011 to only two in 2019. Reasons for the increase in cases in Santo Domingo are unclear, but likely involve domestic rural-to-urban migration and impoverished conditions in affected areas.

**Fig. 1:**
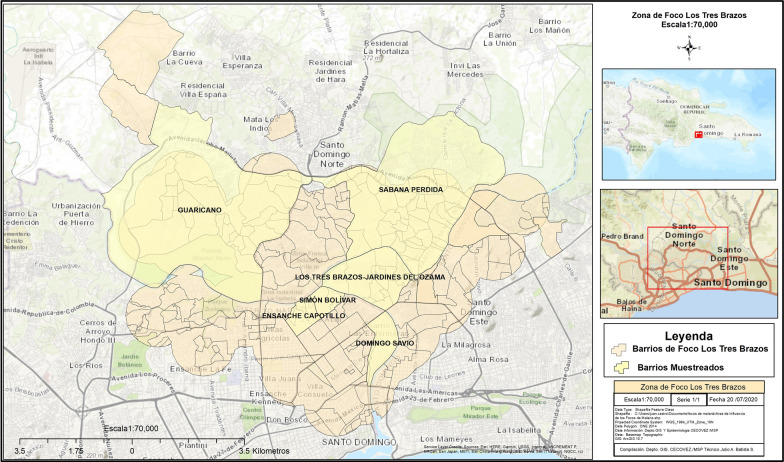
Map of Los Tres Brazos transmission focus (light orange) and sampled neighbourhoods (light yellow), Santo Domingo, Dominican Republic. Image from CECOVEZ

An initial focus of transmission in the capital was the Los Tres Brazos neighbourhood, an area of 93 km^2^ with an estimated population of more than 200,000 people. Situated at the confluence of the Ozama and Isabela rivers, the focus includes parts of the Santo Domingo East and North municipalities and the Distrito Nacional (Fig. 1). Most of the people in the area live in precarious socioeconomic conditions. Since the start of the outbreak in late 2014/early 2015, the country’s national malaria programme (housed within the country’s vector-borne disease agency; Spanish acronym, CECOVEZ) implemented standard anti-malaria interventions in Los Tres Brazos. These included indoor residual spraying (IRS) and distribution of long-lasting insecticidal nets (LLINs), as well as community outreach and enhanced surveillance by community health volunteers [4]. This resulted in, or at least was associated with, suppressed transmission between 2016 and 2018, with only four cases reported in the foci in 2018. However, cases in Los Tres Brazos rapidly increased again in 2019. Up to the week preceding this study (through 12 October 2019), 348 confirmed cases of malaria were detected in the focus—54% of all cases nationwide.

The resurgent and expanding nature of the malaria epidemic in the capital forced CECOVEZ to consider more aggressive strategies. The World Health Organization (WHO) recommends mass drug administration (MDA) for falciparum malaria in certain situations, such as during malaria epidemics and for transmission interruption in areas approaching elimination [7]. Successful MDA requires high population coverage, usually defined as > 80% of the target population [7]. The need for high population coverage and adherence to potentially multiple rounds of MDA underscore the importance of community engagement (CE) [8, 9]. Community engagement strategies typically involve recruiting and training community members to assist with MDA; educating the population about malaria, MDA, and its purpose; countering rumours; providing incentives; and building institutional trust [7, 9]. Rather than another “magic bullet” in the arsenal of anti-malarial interventions [10], community engagement is increasingly understood as a dynamic social process among all stakeholders involved in health interventions [11].

With these issues in mind, this rapid ethnographic assessment (REA) was conducted in Los Tres Brazos to assess malaria knowledge among community members, document attitudes toward potential MDA for malaria, and to generate a formative understanding of health and health care delivery from the community’s perspective. REA is one of several similar research strategies that include rapid assessments, rapid evaluation methods, rapid feedback evaluations, quick ethnographies, focused ethnographies, and real-time evaluations [12, 13]. These methods attempt to quickly generate in-depth, actionable information about sociocultural dimensions of health or disease in order for health programmes to improve delivery and care [13]. Here, the term rapid ethnographic assessment is preferred because it clearly references both the temporal and social science aspects of this methodology. In general, the approach relies on the usual suite of anthropological techniques, such as key informant interviewing, focus group discussions, and observations to characterize local beliefs, perceptions, or practices regarding certain diseases or health issues and then translate that knowledge into appropriate planning and delivery of health interventions [14]. These methods may also be combined with quantitative surveys [15]. Study participants may be sampled theoretically or purposively (in which recruitment is driven by a priori hypotheses). Unlike quantitative survey sampling, which seeks representative samples to make generalizable claims, sampling in qualitative studies seeks to ensure that the data are “saturated,” or that all conceptual categories to explain the data have been identified and exhausted [16]. To do so, a breadth of key informants with personal knowledge, insights, and experiences about the phenomena of interest are recruited until no further theoretical insights emerge.

## Methods

### Study location

Fieldwork took place over seven days in October, 2019 in the Los Tres Brazos focus of Santo Domingo (Fig. 1). The area is comprised of 94 neighbourhoods (barrios). The study was especially interested in community perceptions in areas of relatively high and low transmission, so neighbourhoods were ranked according to malaria incidence in 2019 up to the time of the study. Six neighbourhoods were selected based on transmission intensity (2 high transmission neighbourhoods; 4 medium or low transmission neighbourhoods) and ease of access to the neighbourhoods. The six selected neighbourhoods accounted for 60% of the total malaria cases in the Los Tres Brazos focus up to the week of the study. The neighbourhood with the highest malaria incidence was intentionally over-sampled.

### Recruitment

This study sought to obtain a cross-section of the general adult population within the Los Tres Brazos focus. Inclusion criteria were: age greater than or equal to18 years; speak and understand Spanish; currently live in the study area; have in-depth knowledge about the general community and willingness to talk at length to study interviewers. Purposive sampling was undertaken as needed to obtain more responses from, for example, more men or younger people. Additionally, research assistants were instructed to pay particular attention to persons who said that they would decline participation in MDA and seek more detailed answers from them.

Contact with key informants in selected neighbourhoods was facilitated by existing connections between CECOVEZ and district-level Health Area Directorates (*Direcciones de Áreas de Salud*; DAS) that serve Los Tres Brazos. Typically, a key figure at CECOVEZ would contact gatekeepers at the DAS office, or a community health worker (*promotora*) directly, in the barrio/sub-barrios where sampling was planned. Then, the research assistants would go to that office, meet the gatekeeper, and then be escorted into the community. Once contact with the first few key informants was made, the individuals from the DAS office would then leave the assistants to conduct interviews. Afterwards, research assistants were free to use chain-referral “snowball” sampling after their first interview, go on their own in the community, or ask the DAS office representative or *promotora* for additional assistance in locating another key informant.

The sampling strategy was modified on an ongoing basis based on collected data. At times, the team was directed to collect more interviews among under-represented groups or among those with insights about emerging themes following post-data collection de-briefings.

### Data collection

The study team was comprised of two study coordinators and three research assistants. Two of the assistants were malaria technicians (*evaluadores*) from CECOVEZ who each have more than 20 years of community-based malaria experience in Santo Domingo and had knowledge of the study site. The other assistant was not affiliated with CECOVEZ but had extensive experience in previous research projects with one of the study coordinators (HK).

Research assistants were trained to collect structured interviews, record observations, and hold informal conversations. One of the research assistants already had extensive prior experience with qualitative methods. Each assistant was assigned a target of three structured interviews per day of fieldwork. Interview guide questions (Additional file 1) captured general demographics; general features of daily life, such as work schedules, local organizations, and trusted leaders; knowledge about malaria, including transmission and prevention; sources of health information; trust in the health system and local authorities; and MDA, including existing familiarity with MDA and likely participation in a future MDA campaign. If the participant was unfamiliar with MDA, a brief, standardized definition was provided by the assistant; this definition was developed by CEOCVEZ leadership and through team discussions. Interviews were typically held in participants’ homes, at their place of work, or in public spaces like corner stores (*colmados*) or street corners. Data collection was conducted in the middle-late afternoons, when most people were assumed to be at home. Interviews were not audio-recorded; instead, assistants noted key words and phrases used by the participant in free text boxes following each question in the interview guide.

Second, assistants were trained to record observations as a means to triangulate information collected in interviews. These could span general features of the surrounding area where the interview took place: presence of environmental risks such as mosquito habitats and trash; socioeconomic conditions and behavioural cues of the participant; and population density of the area surrounding the interview site.

Third, assistants recorded informal conversations during each day of fieldwork. These conversations were guided by study objectives and also sought to triangulate findings. For example, if an interview participant described distrust of the local neighbourhood association (*juntas de vecinos*), then the assistant could, afterwards, make brief contact with a passer-by and quickly ask for his or her opinion of the *juntas de vecinos*. These conversations occurred through any informal contact with a general community member, such as on the street, at a bus or moto-taxi stop, or in a *colmado* or beauty salon. The substance of these brief interactions was also recorded in key words and phrases taken by the assistant.

Data quality control was undertaken by study coordinators through serial checks of completed interview forms, assessing completeness of recorded responses, and use of the participant’s own phrasing and vocabulary in recorded responses. Feedback was provided to individual assistants as needed.

### Analysis

Taking a grounded theory approach [17], data analysis took place during team analysis sessions moderated by the study coordinators (HK, KU). The team was encouraged to think broadly about general themes related to malaria, health information, trust in local institutions, and MDA. In this way, assistants applied their own interpretive lens to the data.

Team analysis sessions were held after days 3, 4, and 7 (the final day) of data collection. Each analysis session began with one hour of team discussion moderated by the study coordinators (HK and KU). Then, the assistants wrote a one-page essay in response to several questions put forward by a study coordinator (Additional file 2). These questions were formulated to capture core concepts, themes, and ideas emerging from the data and team discussions. For example, some questions were as follows: why do people trust *promotoras*?; Why do people say that they would participate in MDA? New questions were developed over time as new insights arose from the data or group discussions.

To support their written answers, assistants were asked to elaborate using personal observations and quotations from participants. Afterwards, a study coordinator (HK) typed the written analysis summaries and raw field notes from each assistant into Word documents for content analysis. The study coordinator also took daily notes to track the team’s activities, challenges and areas for improvement, and general impressions and reflections to keep an audit trail.

Finally, participant demographic data and responses to key interview questions from the structured surveys were entered into an Excel spreadsheet for quantitative data analysis. No personally identifying information was recorded in any data collection activity.

### Data summary

Over seven days of site visits, 61 structured interviews and 72 informal conversations were conducted with community members across six sampled neighbourhoods of Los Tres Brazos (Table 1). As shown in Table 2, the structured interview participants (n = 91) were balanced between men (51%) and women (49%). Average age of participants was 44.6 years (range 18–82). Nearly half were married or in a civil union (49%). The average residency time in Los Tres Brazos was 23.6 years (range 3 months to 60 years), indicating a well-established population. Almost half the sample (49%) had no more than a primary education. Common job functions included working as a store owner, being a homemaker, construction, informal work, students, lottery ticket booth sales, carpenters, and salon beauty shop workers.

**Table 1:** Malaria incidence (January 1st–October 12th, 2019) and data collection in sampled neighbourhoods of Santo Domingo

Sampled barrio	Malaria incidence (cases per 1000 persons)	Structured interviews	Informal conversations
Los Tres Brazos	1.9	25	30
Simon Bolivar	1.5	5	7
Capotillo	0.3	4	3
Domingo Sabio	0.6	9	8
Sabana Perdida	0.09	9	11
Guaricano	0.01	9	13
Total	0.4	61	72

**Table 2:** General demographics and key responses among structured interview participants (n = 61)

Variable	n (%)
***Sex***	
Female	30 (49)
Male	31 (51)
***Average age, years (range)***	44.6 (18–82)
***Marital status***	
Married or civil union	30 (49)
Single	31 (51)
***Average residency time in neighbourhood, years (range)***	23.6 (0.25–60)
***Education level, (n = 43 total)***	
Primary	21 (49)
Some high school	9 (21)
High school, completed	8 (19)
Above high school	2 (5)
None	1 (2)
***Occupations***	
Store owner (comerciante) Homemaker (*ama de casa*) Construction Informal work (*chiripero*) Student Lottery ticket booth sales Taxi/public transport driver Public employee Other (barbers, beauticians, mechanics, bakers, cleaners, upholstery, carpentry, retired)	10 (16) 9 (15) 5 (8) 4 (7) 4 (7) 4 (7) 2 (3) 2 (3) 19 (31)

## Results

Based on the analysis sessions, the findings are organized into the following core domains: (1) malaria knowledge, perceptions of healthcare, and other health concerns; and (2) perceptions of anti-malarial MDA, institutional trust, and health messaging.

## Malaria knowledge, perceptions of healthcare, and other health concerns

Overall, most interview participants (64%) affirmed knowledge of malaria (answered “yes” to the interview question, “Do you know what malaria is?”; Additional file 1). In follow-up questions, participants gave a generally accurate description of the disease as transmitted by mosquitoes; causing fever, headache, or body aches; and preventable through the use of mosquito nets, repellent, fumigation, and/or the elimination of breeding sites (*criaderos*). In analysis discussions, research team members remarked that although participants connected malaria with mosquitoes, some confused malaria with dengue, another mosquito-transmitted febrile illness common in the Dominican Republic. For example, when asked how malaria is contracted, a 60 year-old woman in Guaricano said: “From mosquitoes that reproduce in clean water,” likely recalling health messages about dengue (transmitted by *Aedes* mosquitoes that favours small containers, tyres, or natural holes that fill with rain water for breeding).

In fact, participants appeared more familiar with dengue than they did malaria; in analysis discussions, this was said to result from greater exposure to health messaging about dengue than malaria and because of heightened fear of dengue. One assistant said that there are likely political reasons underlying the lack of awareness about malaria. The Dominican Republic is a popular tourist destination, and the country’s political leadership and tourism industry have a vested interest in preventing news about malaria from disseminating beyond transmission areas. Simply put, malaria frightens tourists. There was urgent need to control outbreaks, “before people [in the community] start talking to the press,” one assistant half-joked. All of this was said to contribute to poor dissemination of information about malaria.

Based on interview responses, knowledge about malaria was generally shaped by (1) personal experience or word-of-mouth in past outbreaks, in which a participant and/or his or her family member or neighbour had contracted the disease and dealt with symptoms, care-seeking, and diagnosis first-hand; and (2) health activities led by community health workers (*promotores*). In general, there was a positive perception of *promotores* sponsored by CECOVEZ and the Ministry of Health. *Promotores* are individuals who live within the community and were sometimes said to be the only source of health-related information or initial point-of-care.

Doctors, too, were trusted, although negative experiences in past malaria outbreaks had sown mistrust. For example, some participants recounted how a sick family member or neighbour who had fallen ill was misdiagnosed by a doctor, sent home, and diagnosed later by a health *promotora* after the patient’s condition had worsened. When malaria was suspected (as opposed to other diseases), people often trusted the CECOVEZ field team and community health workers more than doctors, usually based on past experience or overheard anecdotes in which patients were finally diagnosed by a promotora. One 38-year-old man in Los Tres Brazos said in an interview that, “hospitals don’t cure malaria.” Still, many people advised that in general, they would still seek care at hospitals or clinics, likely because they had few options anyway and because medical doctors were still respected for their knowledge: *El que sabe es el medico*—“he who knows is the doctor,” said an older man in an interview. Decisions for seeking healthcare tended to rest with female heads-of-households (*amas de casa*), such as a mother, wife, or grandmother.

**Fig. 2:**
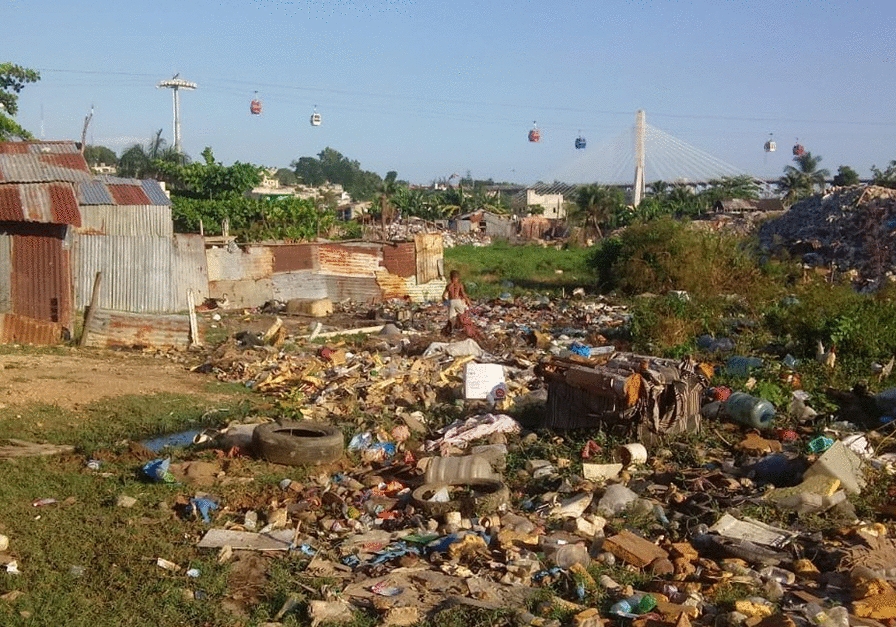
Scene within Las Lila with metro system cable cars overhead and Rosario Sánchez bridge in distance

Malaria was linked to trash, contamination, and inadequate sanitation. People cited the nearby canals (*cañadas*) that were clogged with foul water, emitting a bad odour also said to cause disease (akin to miasma). A few people thought that malaria could be transmitted through water itself. Community residents valued cleanliness, mentioning in interviews that prevention of not just malaria but disease in general could be brought about by attention to hygiene (*limpieza*) of the home and surrounding area. This was in turn linked to environmental contamination in their communities, which surfaced in explanations of disease. “What worries us most is what they will do about this trash here,” said a resident of Las Lila, a particularly marginalized sub-barrio within Los Tres Brazos (Fig. 2). “Sometimes, the trash truck won’t come for [up to] two weeks,” a young man in Sabana Perdida was noted to say in an interview.

Two-fifths (39%) of interview participants said that asymptomatic malaria infection was possible, usually understood from personal experience or word-of-mouth. For example, one woman in Los Tres Brazos said that, “I heard it said that one neighbour here [had] malaria and [he] said that he didn’t feel anything.” Another woman in Capotillo said that “There are many people who are sick and still go about their day as if nothing is wrong.” In an evocative remark by a young man in Los Guandules, asymptomatic malaria was described as something hidden: *Si esta escondido, es algo que esta todavía tapado*—“If it is hidden [not causing symptoms], then it must be something that is covered up,” or undetectable.

The majority (58%) of interview participants said that malaria could not present asymptomatically. “I think that it isn’t possible to not have symptoms, I think [you] must feel something,” said an older man in Los Guandules.

Finally, common health problems cited by community members included gastrointestinal illnesses (vomiting, diarrhoea) as well as infections, such as HIV/AIDS, dengue, the common cold or other viral syndromes known broadly as *gripe*, and papilloma. Chronic diseases mentioned by participants included diabetes, high blood pressure, and cancer.

### Perceptions of anti-malarial MDA, institutional trust, and health messaging

No participant (0%) knew what MDA was. After being provided with a definition of MDA (developed prior to the survey by CECOVEZ leadership; see interview guide in Additional file 1), nearly all (92%) participants said that they would agree to participate, whereas 7% stated they would decline MDA entirely (one response was missing).

The overwhelming support for MDA appears attributable to two key factors: concern for one’s personal health and the desire to help the broader community. “We do not know if tomorrow we [will] fall ill,” said a 55-year-old woman in Los Tres Brazos, expressing a need for prevention. In a way, participation in MDA was one way to circumvent malaria’s unpredictability: “I understand that prevention is better than [trying to] cure the disease,” said a 24-year-old woman in Los Tres Brazos.

Participating in potential MDA was also seen as an act of service. “I would take the medicine to prevent malaria in the neighbourhood,” said a 28-year-old woman who worked in a *banca* selling lottery tickets. Connecting individual participation to community-level benefits was widespread across all sampled communities. In essence, participation in MDA communicated a sense of solidarity and care for the community. To illustrate the vocabulary and phrasing that participants used to communicate this sentiment, exemplary quotes from interviews (accompanied by original Spanish) are compiled in Table 3.

**Table 3:** Interview excerpts that express motivation to participate in MDA as a way to support the general community

Example quotation *Original Spanish*	Participant background
It is a contribution to my community *Eso es un aporte a mi comunidad*	Female, 40 years old, Capotillo
It is a social act that deserves support *Una obra social que merece apoyo*	Male, 59 years old, Los Guandules
For the people of the neighbourhood, because they need it *Por las personas del barrio, porque lo necesitan*	Male, 56 years old, Los Tres Brazos
To help my neighbourhood with this evil disease *Para ayudar a mi pueblo con esa enfermedad mala*	Male, 62 years old, Los Tres Brazos
To cooperate with others *Para cooperar con los demás personas*	Female, 49 years old, Los Tres Brazos
That way, other people in the community won’t get sick *Así no se enfermarían otras personas aquí en el sector*	Male, 21 years old, Sabana Perdida
Because what I want for myself, I want for others *Por que lo que quiero para mi, yo quiero para los demás*	Female, 38 years old, Sabana Perdida
If it benefits the community, I see it as very important *Si es beneficial a la comunidad, lo veo muy importante*	Female, 27 years old, Guaricano

However, several key reasons emerged for the small proportion of those who would refuse participation in MDA. One reason was feeling unsure if the drug administrator was trained competently or doubting whether the medicine was prescribed by a doctor. In one informal conversation, a resident of Los Tres Brazos said that he or she would take the medicine only, “if the public health [team] is trustworthy.” Another in Capotillo said in an interview that, “There are people who won’t take [the medicine], if it isn’t prescribed by a doctor.” An older man in Simon Bolivar said that he, too, would participate only if a doctor prescribed the medicine.

In interviews, another potential reason for declining MDA is that some people would rather decline the MDA in order to observe if others in the community succumb to side-effects. This reflects caution and skepticism towards MDA and underscores the importance people place on their own observations, rather than reassurance by health workers.

Perhaps one of the most obvious hurdles to overcome in a successful MDA is coordinating the MDA with the work schedules of community residents. Work was clearly valued by residents because of precarious economic circumstances. Thus, although they may see participation as a benefit to themselves and their community, their need to earn income may be prioritized above the perceived risk of contracting an otherwise rare disease (Table 4). “I won’t risk a day of [missing] work,” said a 19-year-old man in Los Tres Brazos in an interview; “If I am not working, I would participate,” said a 37-year-old woman in Los Tres Brazos. An older man in Simon Bolivar said in an informal conversation that “If I have the time to do it, I will.” Similarly, a 60-year-old man in Guaricano said in an interview:

Yes, but it depends on the available time [I] have. Many times I am away from the community, but yes, I would take the medicine.

**Table 4:** Profile of interview participants who said that they would reject participation in MDA (N = 4)

Neighbourhood	Gender	Age	Education level	Occupation	Know about malaria	Reason for not participating in MDA
Los Tres Brazos	M	19	Unknown	Barber	No	I work. I must take care of my wife; I cannot risk [losing] a day of work
Los Tres Brazos	F	50	Unknown	Upholstery	Yes	[I] do not trust taking medicine in that way
Simon Bolivar	F	46	Primary	Custodian	Yes	I do not have time
Guaricano	F	42	Primary	Cassava baker	No	I do not like it [the idea of MDA]

Some final points regarding potential refusal to participate in MDA include issues of age and socioeconomic status. Based on informal conversations and observations, young people were said to be generally uninformed about health or disease and did not express much interest in MDA. Alternative modes of communication to spread MDA messages would likely be needed to reach this group, which uses popular social media like WhatsApp, Facebook, Instagram, and YouTube.

Older people, too, expressed unique reasons for hesitating or refusing to participate, such as concern about drug-drug interactions and possible side-effects, because they may already be taking prescribed medicines and conceptualize their health as too fragile for MDA. Another interesting point was that those with upper- and middle-class socioeconomic status may be less inclined to participate in MDA because they have generally had little prior exposure to malaria outbreaks and may feel as though malaria is not a problem for them. To note: this point surfaced in a team analysis session after visiting more well-to-do neighbourhoods.

Neighbourhood associations (*juntas de vecinos*) are widespread throughout the Dominican Republic and are common entry-points for public health programs to begin community outreach [4, 18]. Presumably, community engagement for a future MDA in Los Tres Brazos would also involve working closely with *juntas de vecinos* in the transmission focus. However, most people in this study had a negative opinion of local neighbourhood associations, which were seen as too political and self-serving. “An evil rat,” said one resident in an informal conversation, referring to his *junta de vecinos* in Los Guandules; *ladrones* (crooks), said another in the Sabana Perdida neighbourhood; “they convene us only when they need something,” said another in Simon Bolivar. *Juntas* were also said to be overly political; “the* junta* here resolves problems only for their political party,” said a resident of Simon Bolivar. Finally, the assistants described that some residents seemed to be unsure if their community even had a *junta de vecinos*, reflecting a lack of community-level organization.

Some hypothetical scenarios in which local politics could adversely affect MDA were raised in team analysis sessions. Some politically aligned neighbourhood associations, for example, may seek to capitalize on any large-scale provision of free services in order to gain support among the voting populace and thereby distort MDA campaign messages, such that MDA and ancillary activities appear to be affiliated with a given political party. In contrast, other political parties may seek to undermine MDA activities by spreading misinformation such as, for example, “Political party XYZ is funding the MDA in order to poison this community, because the people here belong to the opposition party.”

However, although trust in *juntas de vecinos* may have been low, some people mentioned certain key figures in their communities who do help to resolve issues. These individuals were often referred to by their nicknames and were said to be available (*dispuesto*) for anything (*cualquier cosa*). Participants also referenced churches as trustworthy sources for information about health and as sources of support in the community. However, sometimes people said that there was simply nobody, or no formal group or organization, in whom they could trust or turn to resolve problems.

Health messaging and health information were closely related to issues of trust as well, carrying additional implications for MDA. Participants cited *promotores*, nearby hospitals or clinics, and even 9-1-1 ambulance staff as sources of health information. Some cited mass communication, such as television and radio, although anecdotally, one research assistant observed that few people actually had televisions (due to their economic circumstances). Even if they did, electricity in the community was said to be unreliable. In one analysis session, one potential idea for spreading messages was identified: the small *gua-guitas* (little trucks) that blast announcements (*perifoneos*) about upcoming events, such as happy hour drink specials at a nearby discotheque or discounted prices for fruits and vegetables.

In addition to spreading information about malaria, word-of-mouth among community members and educative talks (*charlas*) led by *promotores* were seen as important ways to learn about health issues in general. An older man in Simon Bolivar neighbourhood said:

Well, here, whenever there’s a disease, we know about it from others in the neighbourhood talking about it, that’s how it was with dengue.

One woman in Capotillo opined that the best way to get health information was, “from the health *promotores*, because sometimes the news says fake things.” Social media, especially among young participants, were also mentioned as sources of health information.

However, assistants detected a general disconnect between the content of health information that penetrated these communities and the ways in which community members described malaria or disease in general. This tended to be framed in light of the precarious socioeconomic conditions of the community. For example, although many interview participants cited contaminated water, including the nearby Ozama and Isabela rivers, as responsible for disease, they seemed unsure as to exactly how: “There is a disconnect between information channels [how people obtain information] and their social and economic reality,” wrote one assistant during an analysis session.

## Discussion

**Fig. 3:**
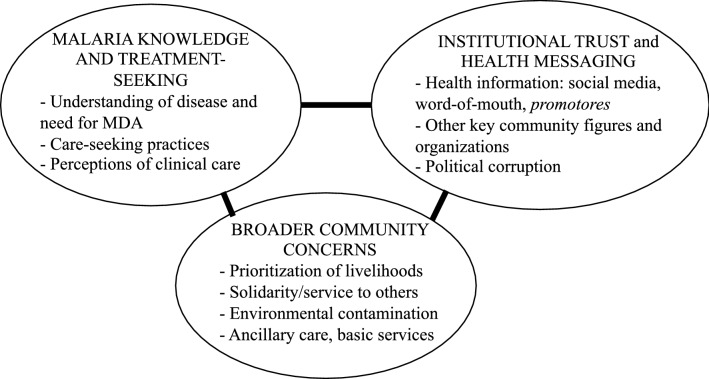
Major factors likely to influence the acceptability of MDA in Santa Domingo

Conducted over seven days, this rapid ethnographic assessment generated contextual understanding of health and healthcare in the Los Tres Brazos focus of Santo Domingo, particularly community perceptions of malaria and MDA as a potential intervention to interrupt transmission and contain an outbreak currently affecting the area. Salient findings and their relevance for MDA planning, implementation and community engagement are summarized in Fig. 3 and Box 1. This work further demonstrates the value of rapid ethnographic assessments to inform community engagement [13, 19] and adds to previous research on motivations and barriers to MDA for malaria on Hispaniola [20] and elsewhere [21–25].

The population appears to have a basic understanding of malaria, trust community health workers (*promotores*), and seem highly motivated to participate in potential MDA. At the same time, challenges to MDA are numerous and require careful consideration. These challenges include careful messaging for MDA; identifying and involving trusted figures in the community; and overcoming issues of mistrust of drug administrators and local political authorities, conflicting work schedules, and de-prioritization of malaria as a problem, especially in the wake of COVID-19.

Awareness of malaria is at least partly attributable to ongoing outbreaks of the disease in the capital. Much of the knowledge about malaria is due to personal experience—of either one’s own malaria infection or that of an immediate family member or acquaintance. Community *promotores* were the source of additional knowledge about malaria. Although many participants who reside in the transmission focus still trust medical professionals—advising that they would seek care at medical centres should they fall ill in general (perhaps because they have few options anyway)—they expressed greater trust in community-based health workers for accurate malaria diagnosis and information. This may be due to previous clinic visits in which malaria was misdiagnosed as other more common febrile illnesses. However, there was an apparent contradiction: participants expressed, on the one hand, mistrust of doctors regarding diagnosis of malaria, but still felt hesitant or skeptical of MDA if they were not assured the medicine was prescribed by a doctor. This discrepancy will need to be carefully considered when conceptualizing the roles of medical professionals and community health workers for potential MDA implementation.

Greater familiarity with dengue may be due to a few reasons. First, dengue is more widespread than malaria, affecting 29 of the country’s 32 provinces. Second, the year 2019 witnessed a dramatic rise in cases nationwide compared to 2018: up to epidemiological 41 (the week of this study), there were 542 cumulative cases nationally, compared to 35 at the same time point in 2018 [26]. Furthermore, annual deaths from dengue have consistently outnumbered deaths from malaria in the Dominican Republic: in 2019, there were 53 deaths from dengue, while there have been fewer than 10 deaths per year from malaria since 2012 [27]. Taken together, these features of dengue, along with public health sensitization campaigns, may have contributed to more awareness and fear of dengue in the general population.

No participant in the study was familiar with the term “mass drug administration” (*administración masiva de medicamentos*). This result is not surprising because the only recent anti-malarial MDA campaign in the country was in 2005 in the El Seibo province, located several hours’ drive from the capital. MDA campaigns against lymphatic filariasis have also taken place in several regions of the country, including annual treatment in the nearby Santo Domingo de Guzman neighbourhood of La Ciénaga from 2004 to 2006 [28]. After providing a formal definition of MDA, nearly all agreed that they would participate. Although desirability bias cannot be excluded, inspection of field notes indicated strong themes of solidarity. Given this finding, one way to ensure high participation in MDA would be to implement MDA as a part of a larger effort to improve community health [24, 25, 29, 30]. Campaign sensitization could use salient idioms and phrases used by participants in this study to build on the idea of participation in MDA as a way to care for and take pride in one’s community (see Table 3 above).

This study revealed low levels of institutional trust in current civic associations and the potential danger for political manipulation of health campaigns. In addition, the COVID-19 pandemic has highlighted the growing trend of medical misinformation. “Fake news” about the pandemic circulates on social media in the country driving demand for ineffective therapies such as hydroxychloroquine [31]. Mistrust of political authorities, counter-narratives to official explanations, and medical malpractice and poor oversight seem to be flourishing. These issues must be proactively addressed to ensure high community participation in MDA. One potential way forward to build trust in affected communities is to conduct MDA in conjunction with other services, such as free medical screening for hypertension and diabetes and donations of children’s school materials, chlorine tablets for home water storage, and iron tablets and prenatal vitamins. Such ancillary activities have been shown to improve participation in MDA in other contexts [24, 25, 30] and would help to align MDA with community health concerns. “Beyond health concerns, [people] see solutions to basic problems, like electricity, potable water, and lack of work as important”, wrote one assistant in an analysis essay; another assistant wrote simply that, “They seek to obtain a better way of life.” Because people also seem attuned to issues of sanitation and pollution, MDA could occur alongside trash clean-ups, elimination of mosquito habitats, bed net distribution, and residual spraying. Medical screenings could be staffed, for example, by local medical and nursing students supervised by resident physicians. The presence of medical doctors on site could also reassure people who may be skeptical of taking medicine without a doctor’s prescription. Ancillary activities like these would signal that MDA is part of a larger concern for community health and would address issues raised by participants in this study.

These findings provide substance to enact key recommendations for community engagement strategies highlighted in other studies [9, 25, 32]. In general, these studies emphasize early formative research about the community; strengthening bonds between communities and MDA program staff; being responsive to community needs and concerns; and fostering shared responsibility. These strategies could become operationalized in this context through, for example, conducting more formative research into themes of community solidarity, social networks, and key community figures; holding community-based educative talks (*charlas*) by trusted *promotores* to raise awareness about malaria; contextualizing MDA as part of a broader effort to improve community health; and incorporating ancillary medical care alongside MDA activities. Even though 64.0% of participants appeared to know about malaria, it will still be crucial to launch educative campaigns that use clear, non-technical language through diverse media and forums. For example, the vocabulary of malaria as being “hidden” (*escondido*) or “covered up” (*tapado*) may be more effective at communicating ideas about asymptomatic infection than biomedical language. Appropriate venues for transmitting information about malaria and MDA could include in-person meetings at community gathering spaces such as churches, youth clubs, *colmados*, and clubs and even through networks of gang leaders or drug dealers in marginalized areas. Involving women’s groups would also be particularly worthwhile because women had considerable decision-making power over health matters in the home. It is important to recall, too, that in this setting, forms of collective organization and mutual aid go back centuries [33]. For example, the *convite* is a form of cooperation among rural neighbours who join together to accomplish tasks [34], while lines of credit (*fiao*) are commonplace at corner store *colmados* for neighbourhood residents in need [35]. To have greater local resonance and generate trust, MDA activities could be modelled on these longstanding cultural practices of mutual aid and reciprocity.

Other important media to consider for transmitting health messages include digital platforms such as YouTube, Facebook, Instagram, and WhatsApp. Word-of-mouth will be important in spreading health information and countering unsubstantiated rumours. Precarious economic circumstances are widespread throughout the transmission focus; a large number of people may lack radios, televisions, and smartphones (or electricity in general). In these cases, it will be important to develop tailored messaging strategies that circulate through reliable intermediaries and other trusted but less formal social networks. It will be necessary to identify, recruit, and supervise these intermediaries, who could spread messages about MDA and counter rumours. Another potentially promising way to spread information about MDA would be through clustered friendship connections [36], which deserve more research in this setting.

Health messages should be harmonized between community and clinical spheres. People can become confused (as they appear already) when they hear from *promotores* that malaria is important and deserves their attention, but are then told by doctors at local clinics that malaria is not a problem. CECOVEZ, community health workers, and clinical staff should all collaborate together on developing a coherent, clear, standardized message in the months leading up to MDA.

Finally, working with local neighbourhood associations (*juntas de vecinos*) may be problematic given the mistrust that community residents appear to have for these institutions. The malaria programme will need to balance the need for collaboration and the risk of appearing too politically aligned or showing favouritism.

Text Box 1 Recommendations for designing and implementing MDA in the Los Tres Brazos focus of Santo Domingo

**Table:** 

Recommendation	Strengths	Challenges
Raise awareness of malaria: Community *charlas* (talks) Social media Public announcements Neighbourhood social networks	Improves general understanding of disease and need for MDA Relies on trusted figures and common ways of receiving information Addresses concerns about side-effects and prescriptions	Harmonization of health messages Clear, comprehensible terminology Inter-sectoral collaboration Rumours and “fake news”
Foster trust between MDA campaign and communities: Community meetings Recruitment of local residents for planning and implementation Trust-building activities (e.g., sharing meals, social events and games)	Increases likelihood for higher participation and acceptability of MDA Strengthens relationship between disease control program and community Creates long-term human bonds for community resilience	Perceived political corruption Failure to account for less obvious social networks and organizations Appearance of patronage and “top-down” imposition of MDA onto community
Fold MDA into broader support of community: Flexibility to account for work schedules Community clean-ups Ancillary care and additional vector-control interventions	Directly addresses concerns of community Fosters trust with MDA campaign Improves efficacy of MDA	Creative strategies needed to accommodate work schedules Inter-sectoral collaboration

## Strengths and limitations

This study was limited by its primary focus on general health and anti-malarial MDA, whereas a traditional malaria knowledge, attitudes, and practices (KAP) survey would shed more light on the spectrum of malaria knowledge, prevention, and treatment in the focus. Therefore, the next step for the research team is to conduct a KAP survey based on a representative sample across a wider segment of the focus. An additional limitation to this study is selection bias. Only individuals who were willing to speak at length about malaria and their communities were purposefully enrolled; those with less interest in MDA, less knowledge about malaria, or harboring mistrust towards health authorities may be under-represented. The definition of MDA did not mention the possibility of side-effects and was given by CECOVEZ-affiliated interviewers, which may have also introduced response bias. The question “Do you know what malaria is?” is also fraught with issues of desirability bias (saying “yes” to a question in biomedical language posed by public health personnel). Furthermore, all three-field assistants were men, which may have influenced rapport with female participants. The shortened timeframe prevented more in-depth training of team members, sampling in other neighbourhoods, obtaining a larger sample, and more time for analysis and reflexivity. Longer-term ethnographic work would provide richer contextual data.

Nonetheless, certain strengths helped address these limitations. First, the team was composed of locally hired assistants, two of which had years of experience in the malaria programme, were familiar with the study site, and had already established rapport with community members. The other assistant was also Dominican and had extensive prior qualitative research experience. Second, the questionnaire was developed at length in team discussions; the team decided that the question “Do you know what malaria is?” would be the most comprehensible to people living in the focus and could be verified by the follow-up questions regarding symptoms and transmission. Third, the built-in analysis sessions allowed the team members to reflect upon their own experiences and observations and interpret emerging themes through shared discussions, which helped identify bias. Fourth, the study included multiple methods to help triangulate findings (structured interviews, informal conversations, personal observations, and team discussions) to confirm or offer alternative explanations. Lastly, one study coordinator (HK) took daily notes as reflexive practice to track sources for bias and how key findings arose in team discussions. Longstanding relationships among all team members and with community members further facilitated team cohesion, lively analysis sessions, and rapport with study participants.

## Conclusion

This rapid ethnographic assessment yielded rich contextual information crucial to evaluating the feasibility of potential anti-malarial MDA. The findings reveal pathways to ensure that MDA be conducted in a socially responsible way.

## Supplementary Information

**Additional file 1. ** Structured interview guide for participants

**Additional file 2. ** Analysis writing session questions

## Data Availability

The dataset used and analysed in this study is available from the corresponding author on reasonable request.

## Abbreviations

CE: Community engagement
CECOVEZ: Centro de Prevención y Control de Enfermedades Transmitidas por Vectores y Zoonosis
DR: Dominican Republic
KAP: Knowledge, attitudes, practices
MDA: Mass drug administration
REA: Rapid ethnographic assessment
WHO: World Health Organization
