# Supplementary material for: Rapid ethnographic assessment for potential anti-malarial mass drug administration in an outbreak area of Santo Domingo, Dominican Republic
Source: Malar J. 2021 Feb 8;20:76. doi: 10.1186/s12936-021-03594-5 (PMC7869078; doi:10.1186/s12936-021-03594-5)
Supplement: Supplementary file 1 — Additional file 1. Structured interview guide for participants [file 12936_2021_3594_MOESM1_ESM.pdf]

Additional file 1

Structured interview guide for participants

Assistant name ___________________
Date and time ____________________
Location ________________________

[After introductions]

1. **Can you describe what kind of work people do here?**

2. **What time do people leave for work, and when do they come back?**

3. **What kind of work do men do? And women?**

4. **What are the biggest health problems in this community?**

5. **Do you know what malaria is?**

6. **How does one contract malaria?**

7. **How does one prevent malaria?**

8. **What are symptoms of malaria?**

9. Do you think it is possible to have malaria and not have symptoms?

10. If you have symptoms of malaria, what would you do?

11. If someone in the home gets sick, who makes the decision to seek care?

12. How does one obtain health information?

13. Do people here trust news and information from doctors or public health?

14. In your opinion, what is the best way people can learn about health problems?

15. **What do people here think about the *juntas de vecinos?***

16. **Who in this community helps to resolve problems?**

17. **Do you know what MDA (mass drug administration) is for malaria? [*If not, note it, and then give the following explanation:*]**

    "MDA consists of giving medicine for malaria to all people in an area at risk for contracting it, regardless of whether [one] has symptoms, to achieve elimination of malaria."
    NOTE THE REACTION OF THE PERSON AFTER READING THEM THE DEFINITION.

18. What do you think about this?

19. Would you participate in MDA, or take the medicine?

20. Why or why not?

First name of participant	Age	Sex	Occupation	Residency time	Civil status	Education level	Telephone number

Key observations – setting of interview: place, other people nearby, type of housing, physical area, trash, mosquito habitats, population density, etc.

<u>Notes from informal conversations</u>:
