# Supplementary material for: Rapid ethnographic assessment for potential anti-malarial mass drug administration in an outbreak area of Santo Domingo, Dominican Republic
Source: Malar J. 2021 Feb 8;20:76. doi: 10.1186/s12936-021-03594-5 (PMC7869078; doi:10.1186/s12936-021-03594-5)
Supplement: Supplementary file 2 — Additional file 2. Analysis writing session questions [file 12936_2021_3594_MOESM2_ESM.pdf]

Additional file 2

Analysis writing session questions

Questions assigned to research assistants for analysis sessions. Assistants gave written responses to each question, using observations and direct quotations from interviews to support their answers. Written essays were then typed into Word documents by study coordinator for content analysis.

Analysis Session 1

1. How would you characterize the level of awareness about malaria?
2. Where are people getting health information?
3. Do people trust the *juntas de vecinos* or others? Who?
4. Do people know what MDA is? After you tell them the definition, how do they react?
5. Do most people say they would participate? Why?
6. What do you think are the biggest challenges to do MDA?

Analysis Session 2

1. In your observations, how was Las Lila different from where you went on the first two days of fieldwork? Can you describe differences in what you saw, what people were doing, how they reacted to you? Why do you think there were these differences?
2. In general [across all areas where you went in LTB], why do people think there is malaria in their community? How do they understand transmission?
3. Why do people trust the *promotoras* for malaria?
4. Why do people say they would participate in MDA?
5. Can you think of reasons why people would not participate in MDA?
6. Who should the malaria program work with, to convince people to do MDA? Why?

Analysis Session 3

1. Do you think people worry about malaria, or are other problems more important to them? What are those problems?
2. How should the malaria program work with the *juntas de vecinos*?
3. Who are the people you think WILL NOT participate in MDA? Can you describe them by occupation, age, or neighborhood? What are reasons why they won't participate?
4. How can we motivate people to participate in the MDA? What can the program do to motivate them?
5. What message should the malaria program use to convince people to do MDA? Why?
6. In general, who or what is most important to the people you have interviewed? What are their hopes or desires? Why are those people and things important to them?
